# Developing a logic model of change for the determinants of parental nurturance in the first 1000 days: A mixed-method study protocol

**DOI:** 10.1371/journal.pone.0258764

**Published:** 2021-10-25

**Authors:** Tessa Goldschmidt, Babatope O. Adebiyi, Nicolette V. Roman

**Affiliations:** The Centre for Interdisciplinary Studies of Children, Families and Society, Faculty of Community and Health Sciences, University of the Western Cape, Cape Town, South Africa; Fondazione Istituto Neurologico Nazionale C Mondino Istituto di Ricovero e Cura a Carattere Scientifico, ITALY

## Abstract

**Background:**

Parents play a key role in providing nurturance and nurturing care to their child during the first 1000 days which is important for optimal child development. Various factors have been found to influence parenting but the contribution of these factors toward parental nurturance within the first 1000 days is not yet known in the South African context. This paper describes a protocol for a project that aims to develop a logic model of change for the determinants of parental nurturance in the first 1000 days in the South African context.

**Method:**

This study will apply a mixed methods approach with a sequential design within an intervention mapping framework. The study will occur in two phases. The first phase will identify the problem, which will be done via a scoping review, a policy review and a needs assessment for parents and stakeholders. This phase will recruit approximately 35 participants (20 parents and 15 stakeholders) for the qualitative component and then approximately 398 participants for the quantitative component. Data will be collected via semi-structured interviews and with questionnaires (Home Observation for Measurement of the Environment Inventory, the Depression and Anxiety Scale, and the Multidimensional Perceived Social Support Scale). Data will be thematically analysed, and the Statistical Package for Social Science (SPSS) will be used to determine descriptive statistics, both of which will inform the development of the model in phase 2. The second phase will be the development of a logic model of change for determinants for parental nurturance in the first thousand days. This phase will consist of one stage- a consensus workshop which will be attended by approximately 20 participants (5 parents, 5 pregnant woman/new mothers, and 10 stakeholders). The data collected in this stage will be thematically analysed and will contribute to the refinement of the model.

**Discussion:**

The first thousand days (FTD) is a period in which rapid growth occurs in all domains of development. If children do not receive sensitive and responsive care in an environment that is conducive for their optimal development, children may not reach their full developmental potential.

## Background

Over the last three decades, scientific evidence suggests that a strong foundation for healthy development in the first thousand days (FTD), is a necessary pre-requisite for wellbeing, economic productivity, and harmonious societies [1, 2]. The FTD (from the time of conception to the age of two) is the most delicate period of child development which lays the foundation for future behaviour, learning, and health [3]. The role of parents in providing nurturance to their child during this period is important for development and to protect children from the effects of adversity by decreasing stress levels and encouraging emotional and cognitive mechanisms [2].

Children develop optimally when their parents (or primary caregiver) nurture their needs. Nurturance has been described as “pervasive attention, emotional investment and behaviour management” by parents or caregivers to foster children’s development [4]. It encourages individuality and self-regulation by responding to the needs of the child [5]. However, for the purpose of this study nurturance will refer to the emotional and physical nourishment and care given to children. Nurturing care is an act of nurturance that starts in pregnancy when mothers sing and talk to the foetus [2]. Children experience nurturing care when they receive adequate nutrition, warm and responsive caregiving, opportunities for early learning, good health, and safety from harm [2]. When children experience reliable care that is sensitive to their needs, they develop a secure attachment to their parent or primary caregiver, which is essential for learning and exploration [6]. Reliable responsive caregiving is therefore essential for optimal development as rapid development occurs in brain structure and capacity [7].

Recent statistics show that many children in South Africa live in unstimulating home environments. According to the General Household Survey of 2018, almost half (46,8%) of children between the age of 0–2 years never read a book or drew (43,1%) with a parent or guardian. The most common form of stimulation was “naming different things” (47.3%), whereas counting (40%) and “talking about different things” (38.8%) with a parent or guardian were less common [8]. These statistics are concerning as findings from a 20-year study show that children who were raised in unfavourable conditions but received high quality early stimulation matured into adults who earned 25% more than their counterparts who did not receive a similar stimulating environment [9].

In order for children to thrive, they need parents to be nurturing—warm, sensitive, and responsive to children’s need [2]. A longitudinal study in America found that the children of mothers who participated in a programme to enhance positive engagement (responsivity and sensitivity) were at a lower risk for behavioural concerns and socio-emotional problems [10]. In another study it was found that parental warmth in childhood was associated with better wellbeing and mental health, and less risky behaviours such as drug use in adulthood [11] which may indicate the benefits of nurturing parenting. In contrast, research also indicates that children who are raised with harsh parenting may be socially withdrawn, mistrustful towards adults or display aggression towards other children because of their fear and negative experiences [2]. While nurturing parenting approaches are ideal, it is often not always the reality due to various factors.

Past research demonstrate that a parent’s ability to be nurturing is influenced factors such as the characteristics of the parent and child (e.g. mental health) and contextual sources of stress and support [12]. In a South African study focusing on the parenting experiences of mothers with mental illness, mothers reported that their parenting abilities such as attending to the needs of their children was compromised due to the side effects of medication [13]. Moreover, Ghebreyesus [14] argues that nurturing care is at risk when children receive inadequate nutrition, limited cognitive stimulation, maltreatment and neglect, disabilities, and exposure to environmental toxins and pollution. These risk factors may cluster in households and children who are exposed to one risk factor may be more vulnerable to multiple risks [14]. Besides the mental wellbeing of parents and the nurturing care that is provided within the home, external factors within a parent’s environment may either hinder or enhance parents’ abilities to nature their child.

Research in the Western Cape Province show that responsive caregiving may be compromised due to elevated levels of community and family violence as well as substance abuse [15, 16]. Almost half of the South African adult population (49.2%) live below the upper bound poverty line [17]. The stress associated with poverty makes sensitive and responsive caregiving difficult, which may negatively affect parent-child interactions [18]. To illustrate, Gozales et al. [19] found that parental warmth and nurturance declined in situations of economic hardship, while harsh and ineffective discipline increased. Furthermore, many children in South Africa live in single parent families, which places greater stress on the parent who primarily support the child. According to Stats SA [8], only 33.8% of children live with both their parents, which is a 6% decrease since 2002 [20]. A study by McConnell, Breitkreuz and Savage [21] found that higher levels of parental social support was associated with lower levels of parenting stress, ineffective parenting and child difficulties. Thus, it is imperative that the social environment in which parents live support nurturing care.

A social environment that enables nurturing care should have policies in place to support parents and child development [2]. Even though South Africa focuses on global health perspectives, nurturing parenting still has a long way to go. Parents in South Africa are entitled to four-months maternity leave and 10 days parental or paternity leave [22], which is vital for nurturing care to occur [2]. Moreover, child support grants are available to assist parents with the costs of the basic needs of children. At the end of March 2019, 12, 445, 000 children between the age of 0 to 17 years received child support grants in South Africa [23]. In addition, although parenting programmes and support is gaining momentum in South Africa, the reality is that many parents still face multiple challenges in nurturing and caring for their children.

The inequalities stemming from South Africa’s complex socio-historical past continue to affect parenting abilities [24] and early childhood development [25]. Many societal factors such as high rates of unemployment, low education levels, unwanted pregnancies, HIV/AIDS, absent fathers, and a culture of violence are prominent in numerous South African communities, which weakens family structures [24] and ultimately influences parenting. Moreover, stress resulting from socio-economic challenges may affect parents’ abilities to provide nurturance and nurturing care to their children [2]. The concept of nurturing care has been prioritised in research as being fundamental for optimal child development, which starts in pregnancy [2].

Although parenting research is an emerging field in South Africa, nurturing parenting research specifically could be considered emerging. Previous research that focused on parents and the FTD in South Africa considered motherhood [26], nutrition [27, 28], interventions [29, 30], and a systematic review on nurturing care [31]. While determinants relating to parenting in terms of child and parents characteristics (e.g. mental health, self-regulation), family structure cultural background, religious beliefs, employment, poverty and financial stress and early childhood development have been highlighted in recent works [32, 33], the deteminants for parental nurturance specifically within the FTD is not yet known. Hence, there is very limited understanding of which determinants influence parental nurturance in the FTD in the South African context. Therefore, there is a need to address this gap by exploring personal and behavioural aspects of parents, coupled with the perceptions of stakeholders in the social environment in which parents raise their children an understanding of determinants of parental nurturance can be unpacked. Thus, this paper describes a protocol to unpack these determinants in the form of a logic model of change.

The study will be guided by the following objectives:

1. To conduct a scoping review on the factors which enhance or hinder parental nurturance in the first 1000 days in previous research.
2. To review the policies which support nurturance and nurturing care in the first 1000 days.
3. To explore how participants understand, describe and apply nurturance with their child and themselves.
4. To explore parents’ and stakeholders’ perceptions of the factors which enhance or hinder nurturance in the first 1000 days.
5. To determine the parenting and wellbeing approaches parents used in the first 1000 days.
6. To determine parents perceived level of social support in the first 1000 days.
7. To develop a logic model of change for the determinants of parental nurturance in the first 1000 days through a concensus workshop.

## Methods

This study will apply a mixed methods approach. A mixed method study was deemed appropriate as it can yield greater insight and understanding of the connections or contradictions between qunatitative and qualitative data, as well as allow for a deeper analysis [34, 35]. The qualitative component is necessary to provide context, to explore participants’ subjective experiences and perceptions, whereas the quantitative component will provide a numeric description of participants’ behaviours, attitudes, or opinion [36] on nurturance and parenting approaches within the FTD.

### Research design

A sequential mixed method design will be used between the two phases of this study, and a concurrent triangulation design will be used in within stages in Phase 1. In the sequential mixed method design qualitative data will be collected followed by quantitative data [37] while the concurrent triangulation design collects both qualitative and quantitative data at the same time [38]. These research designs will be used within an intervention mapping framework. Intervention mapping (IV) is a framework used to guide the development of health promotion programmes [39]. According to Bartholomew et al. [39] the IV process consists of the following six steps with each step entailing different tasks: (1) the development of a logic model based on a needs assessment; (2) create matrices of change objectives by crossing performance objectives (sub-behaviours) with determinants; (3) the selection of theory-based intervention menthods that align with the determinants which turns into strategies or applications; (4) developing an organised programmes by integrating strategies; (5) plan the implementation and sustainability of the program by identifying the needs of the programme users and stakeholders and how their needs will be fulfilled; create a evaluation plan to assess the programme’s effectiveness [39]. The focus on this study will be on the first stage of IV.

This study will therefore be conducted in two phases with different stages.

## Phase 1: Identifying the problem

### Stage 1: Scoping review

A scoping review will be conducted to ascertain the available information on parental nurturance in the FTD. A a scoping review will be used to map the available evidence, to clarify concepts or definitions, to identify key characteristics or factors related to a concept, and to identify gaps in the literature [40, 41]. The question the scoping review aims to answer is: *Which factors enhance or hinder parental nurturance in the first 1000 days in previous research*?

#### Inclusion criteria

Eligibility criteria include studies that are published in or translated in the English language, that focus on parents and/or primary caregivers in the FTD and factors that influence nurturance or nurturing care. Studies will be excluded on the basis of not meeting the inclusion criteria which is studies falling outside of pregancy and the FTD and those that do not focus on nurturance or nurturing care. The period of publications will be from the inception of the database to 2021 as the FTD is considered as an emerging field. There is will be no restriction to geographical areas in which studies were conducted. An extensive search will be conducted to review peer reviewed published and unpublished (grey literature) material such as journal articles, reviews, and theses related to the risk and protective factors of nurturance in the FTD. Data bases such as *Academic Search Complete*, *Ebscohost*, *and Scopus* will be used to source literature. *The following search expression will be used*: (1) parent* OR mother* OR father* OR caregiver, (2) nurtur* OR care OR support (3) first 1000 days OR first two years OR 0 to 24 months, (4) hindrance OR hinder OR barrier* OR challenge enabl* OR enhance OR aid OR improve OR strengthen.

#### Data extraction and analysis

A data extraction tool, based on Peters et al. [42], will be used to organise information according to author, year of publication, country or origin, aim, study population and sample size, methods, and key findings relating to the scoping review question. The findings will provide context and guidance for interviews and the development of the logic model. This stage will take approximately 3 to 6 months.

### Stage 2: Policy review

A policy document review is a method used for exploring the nature of a policy document in order to understand the purpose and essence, and relevance of a document [43]. The aim of the policy review will be to explore which policies support nurturance and nurturing care in the FTD.

#### Data collection

All current policies in South Africa and internationally related to early childhood development and nurturing care in the FTD will be included in the review, which will be sourced from government websites and organisations such as the World Health Organisation and UNICEF.

#### Data analysis

Data will be analysed thematically according to the 6 phases outlined by Clarke and Braun [44]: 1) familiarising yourself with your data by repeatedly reading the data; (2) generating initial codes; (3) searching for themes which involves sorting the different codes into potential themes and organising all relevant coded data extracts within identified themes; (4) reviewing themes by refining themes so that data within themes are coherent; (5) defining and naming themes, which means identifying the essence of what each theme is about (including themes overall) and determining what aspect of the data each theme captures; (6) producing the report which provides a concise, logical and coherent account of the story the data tells within and across themes. The findings will provide guidance for subsequent qualitative data collection and the development of the logic model. This stage will take approximately 3 to 6 months.

### Stage 3: Needs assessment

A needs assessment will be used to assess parenting approaches and parents’ wellbeing, social support, and environmental factors. A needs assessment is used to collect data or information that enhances understanding of the need for programmes or services [45]. The needs assessment will contain both a qualitative and quantitative component. The duration for this stage is estimated to be one year.

#### Research context

This study will take place in the Cape Town Metro. According to Statistics South Africa [46], there were 64 226 birth registrations in the Cape Town Metro in 2018. The Cape Metro has four main health sub-districts namely: Eastern-Khayelitsha, Klipfontein-Mitchells Plain, Northern-Tygerberg, and Southern-Western sub-districts. In these sub-districts, there are a number of Midewife Obstetric Units (MOUs) and clinics that offer antenatal and postnatal care, as well as non-profit organisations (NPO) or non-governmental organisations (NGO) that offer services to parents. Particpants (parents and stakeholders) with diverse demographic backgrounds will be recruited, by the first author, from governmental facilities, NGOs, and NPOs that offer services to parents in low to middle socio-economic income areas within these districts.

### Qualitative component

The qualitative component will explore how parents understand, describe, and apoply nurturance with their child and themselves in the FTD (objective 3) as well as parents and stakeholders perceptions of the factors which enhance or hinder nurturance in the FTD (objective 4)

#### Participants

The first author will purposively recruit participants as they will be selected based on certain criteria [47]. The inclusion criteria are: (1) being a pregnant woman-18 years and older; or (2) being a parent- father or mother, (18 years and older) of a child(ren) between the age of 0 to 2; or (3) being a stakeholder (e.g. community healthcare workers, NGO employees/directors, ECD teachers) who works within an organisation that provides programmes or services in the early childhood development sector for at least 2 years, and more specifically within the FTD. These criteria ensure that the participants will be appropriate for the study as their background allows them to share their perceptions of nurturance within the FTD. Stakeholders perspectives are valued for their experiences of working within the field of early childhood development as they will provide a contextual lens to understand the factors influencing parental nurturance in our context.

#### Data collection

Approximately 35 participants (10 parents- both male and female, 10 pregnant woman and 15 stakeholders) will be recruited for individual interviews. According to Dworkin [48] an adequate sample size for in-depth qualitative interviews is between 5 to 50 participants. The sample size is based on factors such as “the quality of data, the scope of the study, the nature of the topic, the amount of useful information obtained from each participant, and the qualitative method and study design used” [49] or until data saturation has been reached [50]. Semi-structured interviews will be utlised to explore the perceptions of parents and stakeholders. The flexibility of semi-structured interviews enables the researcher to probe the participants on matters that is not necessarily covered in the interview schedule, but is of importance to the participant [51]. Interview schedules (additional file 1) will consist of two sections:(1) demographic information and (2) questions relating to parents’ and stakeholders’ perceptions. These questions will be guided by theory, literature and context. The interviews will be approximately 45 minutes in duration and will be conducted in English, Afrikaans, and isiXhosa if necessary. A Masters student- who is fluent in isiXhosa and English- will be recruited and trained (in conducting interviews and ethics in the research process) to assist with and conduct interviews in isiXhosa to accommodate participants’ participation in their language of choice.

#### Data analysis

The transcribed data will be analysed using Clarke and Braun’s [44] six phases of thematic analysis. The same steps outlined in Phase 1 Stage 2 will be used.

### Quantitative component

The quantitive component will determine parenting and wellbeing approaches used in the FTD (objective 5) and parents’ perceived level of social support in the FTD (objective 6).

#### Participants

Nonprobability convenience sampling will be used to recruit the participants (parents- both male and female) [52]. A sample of 398 participants, as determined by the Yamane [53] Formula (where n = sample size, N = population, e = margin of error), will be recruited for questionnaire completion. Approximately 99 participants per sub-district will be recruited.


$$n=\;\frac{N}{1+N{\left(e\right)}^{2}}\;\text{n}\;\text{=}\;\frac{64\;226}{1+64\;226{\left(0.05\right)}^{2}}\;\text{n}\;=\;397,\;52\;\text{--}\;>\;398$$

#### Data collection

A questionnaire (additional file 1) will be administered face-to-face and online. The questionnaire will collect socio-demographic information and consists of three instruments to assess various determinants of nurturance, namely: the adapted Home Observation for Measurement of the Environment (HOME) Inventory; the Depression, Anxiety, and Stress Scale-21 (DASS-21); and the Multidimensional Perceived Social Support Scale (MPSSS). *The HOME Inventory*, originally developed by Bradley and Caldwell [54], will be used to assess parenting practices in the home environment. The *HOME inventory* measures the quality and quantity of stimulation and support available to a child within the home environment [55]. The respose scale ranges from 0 “does not apply” to 3 “applied to me very much, or most of the time”. The internal consistency of the scale is.80 [56]. *The DASS-21* provides a self-report measure of anxiety, depression, and stress signals [57]. The response scale are given on a 4-point likert scale ranging from 0 “I strongly disagree” to 3 “I totally agree”. The reliability of the scale ranges from.78 to.89 [58]. *The MPSSS*, developed by Zimet, Dahlem, Zimet and Farley [59], assesses social support across domains (family, friends, and significant other). It has a response scale of a 7-point likert scale ranging from 1 “very strongly disagree” to 7 “very strongly agree”. The scale has an internal reliability of.88 [59]. All three scales have cronbach’s alphas above.70 which indicates good reliability [60]. To the authors’ knowledge, the *DASS-21* and *MPSSS* has been used in published studies [61–63] in the South African context, but the use of the *HOME Inventory* has not. Thus, the questionnaire used in this study will be piloted.

Upon receiving ethics clearance, 10% (40 participants) of the sample will be used in a pilot study to test the research protocol, reliability, validity [64], and whether there are any problem areas in the instruments that will be used in the study [65]. The pilot study will be conducted following the same procedures as the main study.

#### Data analysis

SPSS will be used for descriptive statistics to provide information on frequencies, means and standard deviations and prevalence rates with regard to parenting and wellbeing approaches and social support.

## Phase 2: Developing the model

Phase 2 will comprise of the first author developing a logic model of change for the determinants of parental nurturance in the FTD, under the guidance of the third author. The model, which will be based on the PRECEDE model, will addresses the following questions: what is the problem?; what behaviours among the participants contributes to the problem? and what environmental factors contribute to the problem directly or indirectly? These questions will be answered by integrating the findings of Phase 1. The model will be further refined in a consensus workshop. The workshop will adopt a qualitative method.

### Stage 1: Workshop

The purpose of the workshop will be two-fold: to provide participants with feedback of the findings of Phase 1, and present the logic model. The workshop will be held at the University of the Western Cape. It will be an opportunity to engage in member checking and to elicit participants’ opinions on the model. Thus, the overall aim of the workshop is to develop a logic model of change, by refining the initial model according to feedback from participants. This stage and finalising the model may take approximately 2 to 4 months.

#### Participants

10 Parents (5 parents who completed interviews and questionnaires in phase 1 and 5 pregnant/new mothers who completed an interview in phase 1) and 10 stakeholders who participated in Phase 1 will be invited to the workshop.

#### Data collection

The data collection tools that will be needed for the workshop include a laptop and projector for the presentation. A board or news print and markers for the notetaking of participants’ responses, as well as an audio-recorder to capture the session in order for the researcher to capture all the information that was shared. Participants will be asked their opinion on the findings as well as with the model.

#### Data analysis

The data will be transcribed and analysed thematically according to Clarke and Braun [44] in order to reveal any additional themes that will further refine the model.

#### Trustworthiness

The four criteria identified by Guba [66] will be considered in the pursuit of a trustworthy study. The *credibility* (validity) of the study will be ensured by thick descriptions of the context and population, triangulation of data (use of qualiattive and quantitative data sources) [67], frequent debriefing with the supervisor, member checking [68], and peer debriefing [69]. *Transferability* (generalisation) will be ensured by providing information about the researcher as an instrument, the research context, procedures, and participants [70]. *Dependability* (reliability) will be ensured by keeping an audit trail and the examination of analytic memos by the supervisor [70]. *Confirmability* (objectivity) will be achieved through an audit trail and the management of subjectivity [70]. These criteria will be employed in the qualitative component of Phase 1 as well as Phase 2.

#### Self-reflexivity

Self-reflexivity involves self-awareness as researchers ought to acknowledge the personal changes within themselves because of the reseach process and how these changes affected the research process [71]. It requires self-reflection on how researchers’ values, perspectives, and actions can affect data collection and data analysis [72]. The researcher acknowledges her personal demographic background (middle class, coloured female with postgraduate degrees) and that she has no personal experience of parenting. The researcher will remain mindful of this throughout the research process and will ensure that the participants’ voices will be captured and that they will be treated as the experts on this topic. The researcher will record her feelings, thoughts and activities linked to the research process as this aids in developing self-awareness and being cognisant of one’s biases [73]. In addition, the researcher will consult with her supervisor regularly to ensure biases are minimised.

#### Ethics

Ethics clearance (BM20/4/10) has been obtained from the Biomedical Research Ethics Committee at the University of the Western Cape. Upon receiving ethics clearance, this proposal was registered on the National Health Research Database to obtain access to clinics. Permission will therefore be sought from the Western Cape Government: Health’s Research Committee, management of the local clinics and NGOs. Once access to the participants has been granted, the purpose of the research as well as the research process of the study will be explained to the participants. Participants will be encouraged to ask questions to enhance their understanding of their participation in the study. Those that agreed to participate will be provided with an information sheet and they will be asked to complete a written consent form. The participants will be advised on the nature of the study including its aims and that their participation in the study is completely voluntary. Thus participants can withdraw from the study at any time without consequences. The participants will be ensured that all information collected during the study will be held private and will not be disclosed to any parties outside of the study, which ensures their confidentiality. Furthermore, no identifying information will required to participate in the study. The participants will also be assured anonymity as no identifiable information will be used in subsequent reports. In the event that participants should feel distressed arrangements will be made to offer participants counselling. All documentation relating to the study will be stored in a locked cupboard and only the researcher will have access to this information. Audio-files and transcripts will be password protected and stored in a safe place with only the researcher and supervisor having access to it. After 5 years, all data will be disposed of in a manner that it cannot be retrieved. Interview transcripts and questionnaires will be shredded and data files will be permanently deleted from the researcher’s personal computer.

## Discussion

The research question that this study aims to address is: how does a logic model of change explain the determinants of parental nurturance in the first 1000 days? The FTD is a period in which rapid growth occurs in all domains of development [33]. During this period parents and society have the biggest impact on a child’s development. If children do not receive sensitive and responsive care in an environment that is conducive for their optimal development, children may not reach their full developmental potential [2]. In South Africa, many socio-economic factors such as high rates of unemployment and violence are prominent in numerous communities, which weakens family structures [24] and influences parenting. The stress resulting from socio-economic challenges previously mentioned may affect parents’ abilities to provide a nurturing environment for their young children [2]. Therefore, this study is committed to investigating the needs of parents and stakeholders in the Cape Metro. This information will be vital for developing a logic model of change that explains the determinants of parental nurturance in the FTD. The findings of this study may be beneficial for interventions or parenting programmes focused on the FTD as well as a foundation for future interventions or programmes specifically aimed at the FTD in South Africa. Parent support is needed in the South African context where many families live in circumstances that may undermine parenting capacity [74]. Moreover, the findings of this study may be useful for ECD policies in South Africa. Thus, this study will contribute greatly to the growing body of South African literature in parenting within the golden FTD of life.

### Strengths and limitations

We adapted questionnaires that have been validated and used by several researchers for the quantitative part of the study. These questionnaires will be piloted within the study context to enhance the findings of the study. In addition, the mixed method design of this study combines strengths from qualitative and quantitative methods which may enhance the findings of the study. A review of literature published by experts in the areas related to our study was conducted to develop study protocol and the interview schedules for the qualitative sections. The targeted audiences (parents and stakeholders) for the study outcomes will be involved in the problem identification and the development of the model. Possible limitations may include more mothers as participants than fathers as mothers mainly use the sites that will be accessed for data collection. Lastly, the COVID-19 pandemic restrictions may affect access to facilities or organisations for face-to-face data collection. Thus, online methods for data collection may be used.

### Dissemination

Findings from this study will be made available to the participants, government, NGOs and/or NPOs that contributed to the study. Findings will be published in both local and international peer-review journals as well as other forms of journals. In addition, the findings of the study will also be disseminated via various platforms such as conferences, seminars, symposiums, radio as well as social media.

## Supporting information

S1 File Interview schedule and questionnaire. (DOCX)

## Abbreviations

ECD: Early Childhood Development
FTD: First 1000 Days
SPSS: Statistical Package for Social Science
UNICEF: United Nations Children’s Fund
WHO: World Health Organization

## References

[1] Shonkoff J. P., Richter L., van der Gaag J., & Bhutta ZA. An integrated scientific framework for child survival and early childhood development. *Pediatrics* 2012; 129: e460–e472. doi: 10.1542/peds.2011-0366 22218840

[2] World Health Organization UNCF& WBG. *Nurturing care for early childhood development*: *a framework for helping children survive and thrive to transform health and human potential*. Geneva: World Health Organization, 2018.

[3] Hall K., Sambu W., Berry L., Giese S., & Almeleh C. *South African early childhood review 2017*. Cape Town: Children’s Institute, University of Cape Town and Ilifa Labantwana., 2017.

[4] Dishion T. J., & Bullock BM. Parenting and adolescent problem behavior: an ecological analysis of the nurturance hypothesis. In: *Parenting and the child’s world*: *Influences on academic*, *intellectual*, *and social-emotional development (Monographs in parenting series)*. Mahwah: Lawrence Erlbaum Associates Publishers., 2002, pp. 231–249.

[5] Sebire SJ, Jago R, Wood L, et al. Examining a conceptual model of parental nurturance, parenting practices and physical activity among 5–6 year olds. *Soc Sci Med* 2016; 148: 18–24. doi: 10.1016/j.socscimed.2015.11.022 26647364 PMC4714610

[6] Moore, T.G., Arefadib, N., Deery, A. & West S. The first thousand days: An evidence paper. Parkville, Victoria:, 2017.

[7] Cusick S. E., & Georgieff MK. The role of nutrition in brain development: the golden opportunity of the “first 1000 days”. *J Pediatr* 2016; 175: 16–21. doi: 10.1016/j.jpeds.2016.05.013 27266965 PMC4981537

[8] Statistics South Africa. General Household Survey 2018. Pretoria, South Africa, www.statssa.gov.zainfo@statssa.gov.za (2019).

[9] Gertler P., Heckman J., Pinto R., Zanolini A., Vermeersch C., Walker S.,… et al. Labor market returns to an early childhood stimulation intervention in Jamaica. *Science (80-)* 2014; 344: 998–1001. 10.1126/science.1251178 PMC4574862 24876490

[10] Kaminski JW, Perou R, Visser SN, et al. Behavioral and socioemotional outcomes through age 5 years of the legacy for children public health approach to improving developmental outcomes among children born into poverty. *Am J Public Health* 2013; 103: 1058–1066. doi: 10.2105/AJPH.2012.300996 23597356 PMC3698746

[11] Chen Y, Kubzansky LD, VanderWeele TJ. Parental warmth and flourishing in mid-life. *Soc Sci Med* 2019; 220: 65–72. doi: 10.1016/j.socscimed.2018.10.026 30396119 PMC6309475

[12] Belsky J. The Determinants of Parenting: A Process Model. *Child Dev* 1984; 55: 83–96. doi: 10.1111/j.1467-8624.1984.tb00275.x 6705636

[13] Rampou A. M., Havenga Y., & Madumo M. Parenting experiences of mothers living with a chronic mental illness. *Heal SA Gesondheid* 2015; 20: 118–127.

[14] Ghebreyesus TA. Placing Nurturing Care at the centre of global initiatives to improve child health and development. *Early Child Matters Adv early Child Dev* 2018; 17–20.

[15] Rodrigues D, Donita. My kids are then my life: mothering in the context of intimate partner violence, https://open.uct.ac.za/handle/11427/12964 (2014).

[16] Watt M.H., Meade C.S., Kimani S., MacFarlane J.C., Choi K.W., Skinner D., et al. The impact of methamphetamine (‘tik’) on a peri-urban community in Cape Town, South Africa. *Int J Drug Policy* 2014; 25: 219–255. doi: 10.1016/j.drugpo.2013.10.007 24246503 PMC3961551

[17] Statistics South Africa. *Men*, *women and children*: *Findings of the living conditions survey*, *2014/15*. Pretoria, South Africa, 2018.

[18] Diener M.L., Niever M.A. & Wright C. Attachment security among mothers and their young children living in poverty: associations with maternal, child, and contextual characteristics. *Merril-Palmer Q* 2003; 49: 154–182.

[19] Gonzales N. A., Coxe S., Roosa M. W., White R. M., Knight G. P., Zeiders K. H., et al. Economic hardship, neighborhood context, and parenting: Prospective effects on Mexican–American adolescent’s mental health. *Am J Community Psychol* 2011; 47: 98–113. doi: 10.1007/s10464-010-9366-1 21103925 PMC3169846

[20] Hall K. & Sambu W. Demography of South Africa’s children. In: Hall K., Richter L., Mokomane Z. & Lake L (ed) *South African Child Gauge 2018*: *Children*, *Families*, *and the State*: *Collaboration and contestation*. Cape Town: Children’s Institute, University of Cape Town., 2018, pp. 132–137.

[21] McConnell D., Breitkreuz R., & Savage A. From financial hardship to child difficulties: Main and moderating effects of perceived social support. *Child Care Health Dev* 2011; 37: 679–691. doi: 10.1111/j.1365-2214.2010.01185.x 21143271

[22] Africa R of S. Act No. 10 of 2018: Labour Laws Amendment Act, 2018. 42062, South Africa, 2018. Epub ahead of print 2018. 102GOU/B.

[23] Hall K. Income poverty, unemployment, and social grants. In: Shung-King M., Lake L., Sanders D.& Hendricks M (ed) *South African Child Gauge 2019*: *Child and adolescent health*: *Leave no one behind*. Cape Town: Children’s Institute, University of Cape Town., 2019, pp. 221–227.

[24] Department of Social Development. Green Paper on Families: Promoting Family Life and Strengthening Families in South Africa. 34657, South Africa, 2011.

[25] Atmore E. Challenges facing the early childhood development sector in South Africa. *South African J Child Educ*; 2. Epub ahead of print 2012. doi: 10.4102/sajce.v2i1.25

[26] Pentecost M, Ross F. The First Thousand Days: Motherhood, Scientific Knowledge, and Local Histories. *Med Anthropol Cross Cult Stud Heal Illn* 2019; 38: 747–761. 10.1080/01459740.2019.1590825 30945948

[27] Du Plessis LM, McLachlan MH, Drimie SE. What does an enabling environment for infant and young child nutrition look like at implementation level? Perspectives from a multi-stakeholder process in the Breede Valley Sub-District, Western Cape, South Africa. *BMC Public Health* 2018; 18: 1–10. doi: 10.1186/s12889-018-5165-7 29433498 PMC5809892

[28] Pentecost M. The first thousand days: global health and the politics of potential in Khayelitsha, South Africa. Oxford University, 2017.

[29] English R, Peer N, Honikman S, et al. ‘First 1000 days’ health interventions in low- and middle-income countries: Alignment of South African policies with high-quality evidence. *Glob Health Action*; 10. Epub ahead of print 2017. doi: 10.1080/16549716.2017.1340396 28715934 PMC5533118

[30] Tomlinson M., Hartley M., le Roux I. & Rotheram-Borus MJ. The Philani Mentor Mothers Intervention: neighbourhood wide impact on child growth in Cape Town’s peri-urban settlements. *Vulnerable Child Youth Stud* 2016; 11: 221–222. doi: 10.1080/17450128.2016.1220658 29104607 PMC5665390

[31] Work S, Protection C. Nurturing care during the first 1000 days of life: A systematic review LDP Mputle. 2019.

[32] Sanders M.R. & Turner KMT. The importance of parenting in influencing the lives of children. In: Sanders MR& MM (ed) *Handbook of Parenting and Child Development Across the Lifespan*. Springer International Publishing, 2018, pp. 3–26.

[33] D P. Factors Affecting Early Childhood Growth and Development: Golden 1000 Days. *Adv Pract Nurs* 2015; 01: 1–4.

[34] Shorten A, Smith J. Mixed methods research: Expanding the evidence base. *Evid Based Nurs* 2017; 20: 74–75. doi: 10.1136/eb-2017-102699 28615184

[35] Wisdom J. & Cresswell JW. *Mixed methods*: *integrating quantitative and qualitative data collection and analysis while studying patient-centered medical home models*. Rockville, MD: Agency for Healthcare Research and Quality, 2013.

[36] Cresswell JW. *Research Design*: *Qualitative*, *Quantitative and Mixed Methods Approaches*. 4th ed. Thousand Oaks, CA: SAGE, 2014.

[37] Cameron R. A sequential mixed model research design: design, analytical and display issues Publication details Cameron, R 2009, ‘ A sequential mixed model research design: design, analytical and display issues’. *Int J Mult Res Approaches* 2009; 3: 140–152.

[38] Creswell JW, Creswell JD. Research and Design Qualitative, Quantitative and Mixed Methods Approaches. 2018.

[39] Bartholomew Eldredge L. K., Markham C.M., Ruiter R.A.C., Fernádez M.E., Kok G. & Parcel GS G.S. *Planning health promotion programs*: *an intervention mapping approach*. 4th ed. California: John Wiley & Sons, 2016.

[40] Arksey H, O’Malley L. Scoping studies: Towards a methodological framework. *Int J Soc Res Methodol Theory Pract* 2005; 8: 19–32.

[41] Munn Z, Peters MDJ, Stern C, et al. Systematic review or scoping review? Guidance for authors when choosing between a systematic or scoping review approach. *BMC Med Res Methodol* 2018; 18: 1–7. 30453902 10.1186/s12874-018-0611-x PMC6245623

[42] Peters M.D.J., Godfrey C., McInerney P., Baldini Soares C., Khalil H., & Parker D. Chapter 11: Scoping Reviews. In: Aromataris E. & Munn Z (ed) *Joanna Briggs Institute Reviewer’s Manual*. Adelaide, Australia: The Joanna Briggs Institute, https://reviewersmanual.joannabriggs.org/ (2017).

[43] Cardno C. Policy Document Analysis: A Practical Educational Leadership Tool and a Qualitative Research Method. *Educ Adm Theory Pract* 2019; 24: 623–640.

[44] Clarke V. & Braun V. Teaching thematic analysis: Overcoming challenges and developing strategies for effective learning. *Psychologist* 2013; 26: 120–123.

[45] Soriano FI. *Conducting needs assessments*: *a multidisciplinary approach*. 2nd ed. Thousand Oakes, Califronia: SAGE Publications, 2013.

[46] Statistics South Africa. *Recorded live births 2018*. Pretoria, South Africa. Epub ahead of print 2018.

[47] Tashakkori A., & Teddlie C. *Handbook of mixed methods in social & behavioral research*. Thousand Oaks, California: SAGE Publications, 2003.

[48] Dworkin SL. Sample size policy for qualitative studies using in-depth interviews. *Arch Sex Behav* 2012; 41: 1319–1320. doi: 10.1007/s10508-012-0016-6 22968493

[49] Morse JM. Determining sample size. *Qual Health Res* 2000; 10: 3–5.

[50] Saunders B, Sim J, Kingstone T, et al. Saturation in qualitative research: exploring its conceptualization and operationalization. *Qual Quant* 2018; 52: 1893–1907. doi: 10.1007/s11135-017-0574-8 29937585 PMC5993836

[51] Brinkmann S. Unstructured and semi-structured interviews. In: Leavy P (ed) *The Oxford Handbook of Qualitative Research*. New York: Oxford University Press, 2014, pp. 277–299.

[52] Martínez-Mesa J, González-Chica DA, Duquia RP, et al. Sampling: How to select participants in my research study? *An Bras Dermatol* 2016; 91: 326–330. doi: 10.1590/abd1806-4841.20165254 27438200 PMC4938277

[53] Yamane T. *Statistics*: *An introductory analysis*. 2nd ed. New York: Harper and Row, 1967.

[54] Bradley RH, Caldwell BM. The HOME Inventory and family demographics. *Dev Psychol* 1984; 20: 315–320.

[55] Totsika V, Sylva K. The Home Observation for Measurement of the Environment Revisited. *Child Adolesc Ment Health* 2004; 9: 25–35. doi: 10.1046/j.1475-357X.2003.00073.x 32797621

[56] Bradley RH. Children’s home environments, health, behavior, and intervention efforts: A review using the HOME inventory as a marker measure. *Genet Soc Gen Psychol Monogr* 1993; 119: 437–490. 8150270

[57] da Silva H. A., Passos M. H. P. D., Oliveira V. M. A. D., Palmeira A. C., Pitangui A. C. R., & Araújo RCD. Short version of the Depression Anxiety Stress Scale-21: is it valid for Brazilian adolescents? *Einstein (São Paulo)* 2016; 14: 486–493. 28076595 10.1590/S1679-45082016AO3732 PMC5221374

[58] Coker AO, Coker OO, Sanni D. Psychometric properties of the 21-item Depression Anxiety Stress Scale (DASS-21). *African Res Rev* 2018; 12: 135.

[59] Zimet G. D., Dahlem N. W., Zimet S. G., & Farley GK. The multidimensional scale of perceived social support. *J Pers Assess* 1988; 52: 30–41. 10.1080/00223891.1990.9674095 2280326

[60] Tavakol M., & Dennick R. Making sense of Cronbach’s alpha. *Int J Med Educ* 2011; 2: 53–55. doi: 10.5116/ijme.4dfb.8dfd 28029643 PMC4205511

[61] Mahmoud J.S.R., Hall L.A. & Staten R. The psychometric properties of the 21-Item Depression Anxiety and Stress Scale (DASS-21) among a sample of young adults. *South Online J Nurs Res* 2010; 10: 21–34.

[62] Dreyer Z., Henn C. & Hill C. Validation of the Depression Anxiety Stress Scale-21 (DASS-21) in a non-clinical sample of South African working adults. *J Psychol Africa* 2019; 29: 346–353.

[63] Bruwer B., Emsley R., Kidd M. Lochner C. & Seedat S. Psychometric properties of the Multidimensional Scale of Perceived Social Support in youth. *Compr Psychiatry* 2008; 49: 195–201. doi: 10.1016/j.comppsych.2007.09.002 18243894

[64] Arain M., Campell M.J., Cooper C.L. & Lancaster GA. What is a pilot or feasability study? A review of current practice and editorial policy. *BMC Med Res Methodol*; 10. doi: 10.1186/1471-2288-10-67 20637084 PMC2912920

[65] Hassan Z. A., Schattner P., & Mazza D. Doing a pilot study: why is it essential? *Malaysian Fam Physician* 2006; 1: 70–73. PMC4453116 27570591

[66] Guba EG. Criteria for assessing the trustworthiness of naturalistic inquiries. *Educ Commun Technol* 1981; 29: 75–91.

[67] Tracy SJ. Qualitative quality: Eight a “big-tent” criteria for excellent qualitative research. *Qual Inq* 2010; 16: 837–851.

[68] Shenton AK. Strategies for ensuring trustworthiness in qualitative research projects. *Educ Inf* 2004; 22: 63–75.

[69] Marshall C. & Rossman G. *Designing qualitative research*. 5TH ed. Los Angeles: SAGE Publications, 2011.

[70] Morrow SL. Quality and trustworthiness in qualitative research in counselling psychology. *J Couns Psychol* 2005; 52: 250–260.

[71] Palaganas EC, Sanchez MC, Molintas MVP, et al. Reflexivity in qualitative research: A journey of learning. *Qual Rep* 2017; 22: 426–438.

[72] Gerrish K. & Lacey A. *The research process in nursing*. 5th ed. Oxford: Blackwell Publishing, 2006.

[73] McGhee G, Marland GR, Atkinson J. Grounded theory research: Literature reviewing and reflexivity. *J Adv Nurs* 2007; 60: 334–342. doi: 10.1111/j.1365-2648.2007.04436.x 17908129

[74] Ward C., Makusha T. & Bray R. Parenting, poverty and young people in South Africa: What are the connections? In: De Lannoy A., Swartz S., Lake L. & Smith C (ed) *South African Child Gauge 2015*. Cape Town: Children’s Institute, University of Cape Town., 2015, pp. 69–74.

