# Supplementary material for: Developing a logic model of change for the determinants of parental nurturance in the first 1000 days: A mixed-method study protocol
Source: PLoS One. 2021 Oct 25;16(10):e0258764. doi: 10.1371/journal.pone.0258764 (PMC8544825; doi:10.1371/journal.pone.0258764)
Supplement: S1 File — (DOCX) [file pone.0258764.s001.docx]

# **Appendix E: Interview schedule for pregnant women**

**Scetion A: Demographic Information**

| **Age /**ouderdom/ iminyaka |  |
| --- | --- |
| **No. of weeks/months pregnant /**weke/maande swanger /  Inani leeveki okanye iinyanga ezikhulelweyo |  |
| **Gender /** geslag / isini |  |
| **Race/** ras / uhlanga |  |
| **Home language /**huistaal / ulwimi lwasekhaya |  |
| **Area of residence /** woonarea /  indawo ahlala kuyo |  |
| **Highest education level completed/** hoogste akademiese vlak voltooi / ibanga eliphezulu |  |
| **Employment status/** werkstatus **/**  uyaphangelana |  |
| **Occupation/** beroep / umsebenzi |  |
| **Social Grant/** maatskaplike toelaag / yayifumana imali yeGrant |  |

**Section B:**

**Parents understanding, description, and application of nurturance with their child and themselves**

1. What do you understand by the word nurturance or nurturing?

Probe: what do you think is the behaviour of a nurturing parent?

*Wat verstaan u omtrent koestering?*

*Probe: wat is die gedrag van n koesterende ouer?*

Uqonda ntoni ngegama elithi ukukhulisa okanye ukukhulisa?

Probe: yintoni indlela yokuziphatha komzali okhulisa umntwana?

1. How do you nurture and take care of your unborn child?

*Hoe koester en versorg u u se ongebore baba?*

Umkhulisa njani kwaye umgcina njani umntwana wakho ongekazalwa?

1. What do you do to ensure that your baby is healthy?

Probe: what prenatal supplements do you take?

Probe: how many antenatal care visits have you attended?

Probe: how far along were you when you went for your first antenatal care visit?

*Watter stappe neem jy om te verseker jou is baba gesond?*

*Probe: watter voorgeboortelike aanvullings neem u?*

*Probe: hoeveel voorgeboorte sorg besoeke het u bygewoon?*

*Probe: hoe lank was u swanger voor die eerste voorgeboorte sorg besoek?*

Wenza ntoni ukuqinisekisa impilo yomntwana wakho?

Probe: uthatha ziphi izinto zokuncedisa ekukhulelweni?

Probe: Bangaphi utyelelo lokugcina abantwana abakhulelwe?

Probe: ubumde kangakanani xa ubuye kutyelelo lwakho lokhathalelo lokuqala lwebele?

1. What do you do to ensure that you are healthy?

Probe: what do you do to relax when you feel stressed for example?

*Wat doen u om u se gesondheid te verseker?*

*Probe: wat doen u om u spanning te verlig?*

Wenza ntoni ukuqinisekisa ukuba usempilweni?

Probe: Wenza ntoni ukuze uphumle xa uziva uxinzelelo?

1. How do you bond with your baby?

Probe: how often do you talk, sing, and / or read to your baby?

*Hoe vorm u ‘n band met u ongebore baba?*

*Probe: hoe dikwels praat, sing, en lees u vir die baba?*

udibana njani nomntwana wakho?

Probe: Uthetha kangakanani, ucula, kwaye ufundela umntwana wakho?

**Factors which enhance or hinder parental nurturance**

1. How are you preparing for parenthood?

Probe: how are you getting information about parenting or how to raise a child?

*Hoe berei u voor vir ouerskap?*

*Probe: watter stappe neem u om meer inligting te kry oor hoe om n baba groot te maak?*

Ulungiselela njani ukuba ngumzali?

Probe: ulufumana njani ulwazi malunga nokuba ngumzali okanye ukhulisa umntwana njani?

1. Describe the relationship between you and the father of your child?

Probe: how does he support you?

Probe: how is he involved financially or emotionally?

*Beskryf die verhouding tussen u en die kind se pa..*

*Probe: watter ondersteuning bied hy aan?*

*Probe: hoe is hy finansi*eël *en emosioneel betrokke?*

Chaza ubudlelwane phakathi kwakho notata womntwana wakho?

Probe: ukuxhasa njani?

Probe: ubandakanyeka njani kwezemali okanye kwimvakalelo?

1. What do you find difficult or challenging during your pregnacy journey?

*Watter uitdagings het u ervaar tydens swangerskap?*

Yeyiphi imiceli mngeni oye wahlangabezana nayo ngexesha lokukhulelwa kwakho?

1. How does your family and friends support you?

*Watter ondersteuning verkry u van familielede en vriende?*

ikukxhasa njani intsapho yakho kunye nabahlobo bakho?

1. What services / support structures are available in your community to assist you as a pregnant woman?

Probe: can you rely on your neighbours, or religious organisations help when you are in need? Can you share an example of you being helped in the past (in relation to parenting support)?

*Watter dienste of opleidingstrukture is beskikbaar vir swangervroue in u gemeenskap?*

*Probe: Kan u staatmaak op bure of godsdienstige organisasies in n tyd van nood? Verskaf ‘n voorbeeld van hoe iemand hulp verleen het in die verlede (in verband met ouerskap ondersteuning)?*

Zeziphi iinkonzo okanye imibutho yenkxaso ekhoyo ekuhlaleni ekuncedeni njengomfazi okhulelweyo?

Probe: Ngaba unokuthembela kubamelwane bakho, okanye imibutho yezenkolo iyanceda xa uswele? Ngaba ungabelana ngomzekelo wakho wokuncedwa kwixesha elidlulileyo (ngokunxulumene nenkxaso yomzali)?

1. What kind of services or training do you feel is needed in your area for pregnant women?

*Watter dienste of opleiding word in u area benodig vir swanger vroue*?

Loluphi uhlobo lweenkonzo okanye zoqeqesho ocinga ukuba luyafuneka kwindawo yakho kubantu basetyhini abakhulelweyo?

1. *If employed:* In which way does your workplace support you as pregnant woman?

*If unemployed*: How can workplaces support pregnant women?

Probe: for e.g. does your company have any policies regarding breastfeeding or child care?

*Indien in diens geneem: Watter ondersteuning bied u werkgewer aan in verband met swanger vroue?*

*Indien werkloos: Hoe kan werkgewers swanger vroue ondersteun?*

*Probe:* *Beskik jou werkgewer oor n beleid wat borsvoed of kindersorg behels*?

Ukuba uqashiwe: Ngaba yeyiphi indawo ekusetyenzelwa kuyo ekuxhasa njengebhinqa elikhulelweyo?

Ukuba awusebenzi: indawo yokusebenza inokuxhasa njani abafazi abakhulelweyo?

Probe: Umzekelo inkampani yakho okanye indawo osebenayo inemigaqo-nkqubo emalunga nokuncancisa okanye ukhathalelo lwabantwana?

1. In which way does the government support you as a parent?

*Watter ondersteuning bied die regering aan vir ouers?*

Urhulumente ukuxhasa njani wena njengomzali?

1. In your opinion, how can the government better support pregnant women?

*In jou opinie, hoe kan die regering beter ondersteuning aanbied vir swanger vroue?*

Ngokoluvo lwakho, urhulumente angabaxhasa njani ngcono abantu ababhinqileyo abakhulelweyo?

1. What information or tips have you received on parenting or how to care of your baby at your antenatal care visits?

*Watter inligting of wenke het u gekry oor ouerskap of kindersorg by voorgeboortesorg besoeke?*

Luluphi ulwazi okanye iingcebiso osele uzifumene malunga nokuba ngumzali okanye indlela yokumkhathalela umntwana wakho xa undwendwela?

1. Every parent wants to be the best parent they can be. What do you think, if anything, would make you a better parent?

*Alle ouers streef daarna om die beste ouer te wees. Wat in u mening, indien enigiets, sal u ‘n beter ouer maak?*

Wonke umzali ufuna ukuba ngowona mzali ubalaseleyo banokuba nguye. Yintoni ocinga ukuba ikho, engakwenza ukuba ube ngumzali ongcono?

# **Appendix F: Parents Interview Schedule**

**Scetion A: Demographic Information**

| **Age of parent and child /** ouderdom van ouer en kind / Iminyaka yomzali nomntwana |  |
| --- | --- |
| **Gender of parent and child /** Geslag van ouer en kind /  Isini somzali nomntwana |  |
| **Race /** ras / uhlanga |  |
| **Home language /**huistaal / ulwimi lwasekhaya |  |
| **Area of residence /**woonarea / indawo ahlala kuyo |  |
| **Highest education level completed/** Hoogste akademiese vlak voltooi / ibanga eliphezulu |  |
| **Employment status** / werkstatus / uyaphangelana |  |
| **Occupation /** beroep / umsebenzi |  |
| **Social Grant/** maatskaplike toelaag / yayifumana imali yeGrant |  |

**Section B:**

**Parents understanding, description, and application of nurturance with their child and themselves**

1. What do you understand by the word nurturance or nurturing?

Probe: what is the behaviour of a nurturing parent?

*Wat omtrent verstaan u van koestering?*

*Probe: Wat is die gedrag van n koesterende ouer?*

Uqonda ntoni ngegama elithi ukukhulisa okanye ukukhulisa?

Probe: Yintoni indlela yokuziphatha komzali okhulisa abantwana?

1. How do you nurture and take care of your child?

*Hoe koester en versorg u vir jou kind?*

Umkhulisa njani kwaye umkhathalela njani umntwana wakho?

1. How do you take care of yourself so that you are able to parent to the best of your ability? Probe: what do you do to relax?

*Hoe kyk u na jouself om die beste moontlike ouer te wees in jou vermoë?*

*Probe: what do you do to relax? Wat doen u om te ontspan?*

Ngaba uzinakekela njani ukuze ube ngumzali ngokusemandleni akho?

Probe: Wenza ntoni ukuze uphumle?

1. What is your usual daily routine with your child?

*Wat is jou daaglikse roetine met u kind?*

Yeyiphi indlela yakho yesiqhelo imihla ngemihla kunye nomntwana wakho?

1. How do you ensure the health of your child?

Probe: During your pregnancy, what did you do to ensure your baby was healthy?

Probe: how many antenatal care visits did you attend?

Probe: has your child received all his/her vaccinations?

Probe: How often or when do you take your child to the clinic or doctor?

*Hoe verseker u die gesondheid van jou kind?*

*Probe: Tydens swnagerskap, wat het u gedoen om die gesondheid te verseker jou baba?*

*Probe: Hoeveel voorgeboortesorg besoeke het u afgelê?*

*Probe: Het u kind al sy/haar inentings ontvang?*

*Probe: Hoe gereeld of wanneer besoek u kind n kliniek of dokter?*

Uyiqinisekisa njani impilo yomntwana wakho?

Probe: Ngexesha lokukhulelwa kwakho, wenza ntoni ukuqinisekisa ukuba umntwana wakho usempilweni?

Probe: Bangaphi abatyeleleyo abakhathaleleyo abakhulelweyo?

Probe: Ngaba umntwana wakho ulufumene lonke ugonyo lwakho?

Probe: Uhamba kangaphi okanye umthatha nini umntwana wakho ekliniki okanye kwagqirha?

1. Describe the relationship between you and your child?

Probe: how do you bond with your child?.

Probe: how much time do you usually spend with your child?

*Hoe sal u die band tussen jouself en jou kind beskryf?*

*Probe: Hoe verseker u n hegte band met die kind?*

*Probe: normaalweg, hoeveel tyd spandeer u met die kind?*

Chaza ubudlelwane phakathi kwakho nomntwana wakho?

Probe: Uthandana njani nomntwana wakho?

Probe: Lingakanani ixesha oqhele ukuchitha ngalo nomntwana wakho?

1. In what way do you expresss your emotions toward your child?

Probe: do you have difficulty expressing your emotions to your child or talking about feelings? Why do you think this is the case?

*Hoe stel u, u emosies ten toon teenoor u kind?*

*Probe: Sukkel u om jou emosies te toon teenoor jou kind of om dit te bespreek?*

Ubonisa njani uvakalelo lwakho kumntwana wakho?

Probe: Unobunzima ekubonakaliseni imvakalelo yakho kumntwana wakho okanye uthetha ngeemvakalelo? Kutheni ucinga ukuba kunjalo?

1. How often do you talk, play, and sing to your child?

Probe: How often did you talk, sing, or read to your child during your pregnancy?

Probe: What do you talk to your child about?

Probe: What do you play?

*Hoe gereeld praat, speel en sing jy met jou kind?*

*Probe: Hoe gereeld het u tydens swangerskap met u kind gepraat, gesing of lees?*

*Probe: Waaroor praat u met jou kind?*

*Probe: Watter speletjies speel jul?*

Uthetha kangakanani, udlala kwaye uculele umntwana wakho?

Probe: Uthethe kangaphi, ucula, okanye ufundela umntwana wakho ngexesha lokukhulelwa kwakho?

Probe: Uthetha ntoni nomntwana wakho?

Probe: Udlala ntoni?

1. How often do you read with your child?

Probe: When did you start or what age will you start?

Probe: At what age do you think parents should start reading to children and why?

*Hoe gereeld lees u met jou kind?*

*Probe: Watter ouderdom het/gaan u begin?*

*Probe: Op watter ouderdom dink jy ouers vir hul kinder moet begin lees en hoekom?*

Ufunda kangaphi kunye nomntwana wakho?

Probe: Uqale nini okanye uza kuqala iminyaka emingaphi?

Probe: Ucinga ukuba abazali bafanele baqale ukufundela abantwana ngasiphi iminyaka kwaye ngoba?

1. What do you do together to have fun during playtime?

*Wat doen jul saam vir pret tydens speeltyd?*

Probe: Nenza ntoni kunye ukuze nizonwabise ngexesha lokudlala?

1. What do you do to ensure the safety of your child?

*Wat doen jy om die veiligheid van jou kind te verseker?*

Wenza ntoni ukuqinisekisa ukhuseleko lomntwana wakho?

**Factors which enhance or hinder parental nurturance**

1. If you think back to your own childhood, how does the way you raise your child differ from the way in which you were raised?

Probe: What do you do differently in comparison to how your parents raised you? Please share an example.

*As jy terugdink na jou kinderdae, hoe verskil die wyse waarop jy grootgemaak was teenoor hoe jy jou kind grootmaak?*

*Probe: Wat doen jy verskillend in vergelyking teenoor jou ouers? Gee n voorbeeld.*

Ukuba ucinga emva ebuntwaneni bakho, ingaba indlela okhulise ngayo umntwana wakho yahluke njani kwindlela okhuliswe ngayo?

Probe: Wenza ntoni ngokwahlukileyo xa kuthelekiswa nendlela abakhulise ngayo abazali bakho? Nceda wabelane ngomzekelo.

1. How did you prepare for parenthood?

Probe: did you read parenting books? Attend antenatal classes?

*Hoe het jy vir ouerskap voorberei?*

*Het u ouerskap boeke gelees? Voorgeboortesorg klasse bygewoon?*

Ubulungiselela njani ukuba ngumzali?

Probe: Ukhe wazifunda iincwadi zokuba ngumzali? Ukuya kwiiklasi zaphambi kokubeleka?

1. Why should parents be present and involved in their child’s life?

*Waarom moet ouers teenwoordig en betrokke wees in die kind se lewe?*

Kutheni abazali kufuneka babekhona kwaye babandakanyeke kubomi bomntwana wabo?

1. In what way does your family and friends support you?

*Op watter wyse ondersteun jou familie en vriende jou?*

Inokuxhasa njani usapho lwakho kunye nabahlobo bakho?

1. How would you describe the relationship between you and the father of your child?

Probe: how does he support you and the child?

Probe: how is he involved financially or emotionally with the child?

*Hoe sal jy die verhouding tussen jouself end die kind se pa beskryf?*

*Probe: Hoe ondersteun hy jul en die kind?*

*Probe: Hoe is hy finansieel of emosioneel betrokke by die kind?*

1. What are some of the challenges of parenthood that you encountered so far?

*Watter uitdagings van ouerskap het jy so ver ervaar?*

Yeyiphi eminye imiceli mngeni yokuba ngumzali okhe wahlangana nayo ukuza kuthi ga ngoku?

1. What services / support structures are available in your community to assist parents?

Probe: can you rely on your neighbours, or religious organisations help when you are in need? Can you share an example of you being helped in the past (in relation to parenting support)?

*Watter dienste/ondersteunings strukture is beskikbaar vir ouers in jul gemeenskap?*

*Probe: kan u op u bure, of Godsdienstige organisasies staatmaak vir hulp as u in nood is? Kan u ‘n voorbeeld gee van ‘n tyd wanneer jy in die verlede hulp verkry het (in terme van ouerskap ondersteuning)?*

Zeziphi iinkonzo / izibonelelo zenkxaso ezikhoyo ekuhlaleni ekuncedeni abazali?

Probe: Ngaba unokuthembela kubamelwane bakho, okanye imibutho yezenkolo iyanceda xa uswele? Ngaba ungabelana ngomzekelo wakho wokuncedwa kwixesha elidlulileyo (ngokunxulumene nenkxaso yomzali)?

1. What kind of services or training do you feel is needed in your area for parents of young children (0-2years)?

*Watter dienste of opleiding glo u word benodig in jul area vir ouers van jongkinders (0-2 jaar oud)?*

Loluphi uhlobo lweenkonzo okanye zoqeqesho ocinga ukuba luyafuneka kwindawo yakho kubazali babantwana abancinci (iminyaka eli-0-2)?

1. *If employed:* In which way does your workplace support you as a parent?

*If unemployed*: How can workplaces support parents of young children?

Probe: for e.g. does your company or workplace have any policies regarding breastfeeding or child care?

*Indien in diens geneem: Waarmee ondersteun jou werkgewer u as n ouer?*

*Indien werkloos: Hoe kan werksplekke ouers ondersteun met jongkinders?*

*Probe: Beskik jou werkgewer oor n beleid wat borsvoed of kindersorg behels?*

Ukuba uqeshiwe: Ngaba indawo osebenza kuyo ikuxhasa njengomzali?

Ukuba akasebenzi: Indawo yokusebenza inokuxhasa njani abazali babantwana abancinci?

Probe: umzekelo ngaba inkampani yakho okanye indawo osebenayo inemigaqo-nkqubo emalunga nokuncancisa okanye ukhathalelo lwabantwana?

1. In which way does the government support you as a parent?

*Op watter wyse ondersteun die regering jou as n ouer?*

Urhulumente ukuxhasa njani wena njengomzali?

1. In your opinion, how can the government better support parents of young children?

*In jou opinie, hoe kan die regering ouers met jong kinders ondersteun?*

Ngokoluvo lwakho, urhulumente angabaxhasa njani ngcono abazali babantwana abancinci?

1. What information or tips did you receive on parenting or how to care of your child at your antenatal care visits?

*Watter inligting of wenke het u oor ouerskap of kindersorg by voorgeboortesorg besoeke ontvang?*

Loluphi ulwazi okanye iingcebiso ozifumeneyo malunga nokuba ngumzali okanye indlela yokumkhathalela umntwana wakho kutyelelo lwakho lokhathalelo lwaphambi kokubeleka?

1. Every parent wants to be the best parent they can be. What do you think, if anything, would make you a better parent?

*Alle ouers streef daarna om die beste ouer te wees. Wat in jou mening, indien enigiets, maak van jou ‘n beter ouer?*

Wonke umzali ufuna ukuba ngowona mzali ubalaseleyo banokuba nguye. Yintoni ocinga ukuba ikho, engakwenza ukuba ube ngumzali ongcono?

# **Appendix G: Stakeholders Interview schedule**

| **Age /** *ouderdom /* iminyaka |  |
| --- | --- |
| **Gender /** *geslag /* isini |  |
| **Race /** *ras /* uhlanga |  |
| **Home language/** *huistaal* / ulwimi lwasekhaya |  |
| **Area you work in/** *werksarea /* Indawo osebenza kuyo |  |
| **Highest education level completed /** *hoogste akademiese vlak voltooi /* ibanga eliphezulu |  |
| **Occupation /** *beroep /* umsebenzi |  |

**Section B: Which factors enhance or hinder parental nurturance?**

1. What is your understanding of parental nurturance and nurturing care?

*Wat verstaan jy van ouerlike versorging en koestering?*

Uyintoni ukuqonda kwakho ngendlela yokukhulisa nokukhulisa abantwana?

1. From your observations, in which way are children being nurtured and cared for?

*van jou waarneemings, in watter manier word kinders gekoester en versorg?*

Ukusuka kwimigqaliselo yakho, ingaba abantwana bakhuliswa kwaye bakhathalelwe ntoni?

1. In your position, in which way do you help parents (or primary caregivers or families) and early childhood development in our context?

Probe: how do you promote nurturing care?

*In die posisie wat jy jouself bevind, op watter manier help jy ouers (of families) en vroeër kinderontwikkeling in ons konteks?*

*Probe: op manier bevorder u versorging?of verleen jy help op ouers?*

Kwisikhundla sakho, yeyiphi indlela obanceda ngayo abazali (okanye abagcini babantwana abanonophela okanye iintsapho) kunye nophuhliso lwabantwana abancinci kwimeko yethu?

Probe: uyikhuthaza njani ikhulisa inkathalo?

1. From your understanding and experiences, how do workplaces support parents?

*Van jou begrip en ervaaring, hoe ondersteun werkplekke ouers?*

Ngokuqonda kwakho kunye namava akho, iindawo zokusebenza ziyabaxhasa njani abazali?

1. In which way, do you think, do community members support parents in nurturing their children?

*Op watter manier dink jy ondersteun gemeenskaplede ouers om hulle kinders te koester*?

Ucinga ukuba yeyiphi indlela abahlali abaxhasa ngayo abazali ekukhuliseni abantwana babo?

1. In which way can workplaces better support parents of young children?

*Op watter manier kan werkplekker ouers van jong kinders better ondersteun?*

Kungayiphi indlela iindawo zomsebenzi ezinokuthi zixhase ngcono abazali babantwana abancinci?

1. In which way does the government support parents within the first 1000 days?

Probe: In terms of health care, finacial support, and policies.

*Hoe ondersteun die regering ouers in die eerste 1000 dae?*

*Probe: in terme van gesondheids dienste, finansies, en polis?*

Urhulumente ubaxhasa njani abazali kwiintsuku ezili-1000 zokuqala?

Probe: Ngokumalunga nokhathalelo lwempilo, inkxaso yemali, kunye nemigaqo-nkqubo.

1. What services or organisations offer support to parents in the first 1000 days?

*Watter dienste of ogranisasies bied ondersteuning aan ouers in die eerste 1000 dae?*

Zeziphi iinkonzo okanye imibutho enika inkxaso kubazali kwiintsuku ezili-1000 zokuqala?

1. In your experience, what are the challenges when it comes to parents’ caring for and nurturing their children?

Probe: In terms of finances, parenting skills, information etc.

*In jou ervaaring, wat dink jy is die uitdagings wat ouers ervaar as dit by kinder-koestering en versorging kom?*

*Probe: in terme van finansies, ouerse se vaardighede, informasie ens.*

Kumava akho, yeyiphi imiceli mngeni xa kuziwa kubazali ukukhathalela nokukhulisa abantwana babo?

Probe: Ngokwezezimali, izakhono zobuzali, ulwazi.

1. In terms of community life, what are the factors that may directly or indirectly influence a parent’s ability to provide a nurturing environment?

*In terme van die gemeenskap, watter faktore beinvloed, direk of indirek n ouer’s se vermoë om ‘n koestering omgeving vir hul kind te bied?*

Ngokommandla woluntu, zeziphi izinto ezinokubangela ngqo okanye ngokungathanga ngqo kumzali ukuba akwazi ukubonelela ngendawo ekhulisayo?

1. In your opinion, how can parents be better supported in their parenting abilities?

*In jou opinie, hoe kan ouers beter ondersteun word in hulle our ouerskap?*

Ngokoluvo lwakho, abazali banokuxhaswa njani ngcono kubuchule babo bokuba ngumzali?

1. In which way would you, as a stakeholder, like to be supported so that you can better fulfill your role in society?

Probe: in which way can people in support your organisation?

Probe: in which way can the government better support you?

*In watter manier wou jy as ‘n belanghebbendes ondersteun word so dat u u se rol beter kan vervul in die sameleweng?*

*Probe: in watter manier kan kan mense jul organisasie ondersteun?*

*Probe: in watter manier kan die regering jul organisasie ondersteun?*

Yeyiphi indlela wena njengomntu ochaphazelekayo, onqwenela ukuxhaswa ukuze uphumeze indima yakho kuluntu?

Probe: bangayifumana njani indlela abantu abawuxhasa ngayo umbutho wakho?

Probe: urhulumente angakuxhasa ngantoni?

# **Appendix H: Questionnaire for parents**

| **The development of a logic model of change for determinants of parental nurturance in the first 1000 days**  *Izinto ezinefuthe ekukhuliseni kwabazali kwintsuku eziyi1000 zokuqala kobomi bomntwana: Ukuphulisa imodel yotshinitsho*  Bepalende faktore van ouerlike versorging in die eerste 1000 dae: Die ontwikkeling van n logika van verandering | | | | | | | | | | | | | | | | | | |
| --- | --- | --- | --- | --- | --- | --- | --- | --- | --- | --- | --- | --- | --- | --- | --- | --- | --- | --- |
| **Section A: Demographics** | | | | | | | | | | | | | | | | | | |
| **Personal information** | | | | | | | | | | | | | | | | | | |
| **Gender /** *isini /* geslag | - Male /*Ndoda/* manlik | | | | | - Female */ umntu ongumama/* vroulik | | | | | | | | | | | | |
| **Age /** *Iminyaka/* ouderdom |  | | | | | | **Age of your child/** *Iminyaka yomntwana wakho/* Ouderdom van kind: | | | | | | | | | | | |
| **Race/** *Uhlanga***/** ras | Black | | Coloured | | | White | | | | | | Indian | | | Other | | | |
| **Home language/** *Ulwimi lwasekhaya/ huistaal* | English | | Afrikaans | | | IsiXhosa | | | | | | isiZulu | | | Other | | | |
| **Highest Education Level Completed/** *ibanga eliphezulu/* hoogste onderwysvlak voltooi |  | | | | | | | | | | | | | | | | | |
| **Employment status/** uyaphangelana**/** werkstatus | - Employed / *ndiqeshiwe/*werksaam | | | | | | | | | | - Unemployed / *ndiqeshiwe/*werkloos | | | | | | | |
| **What is your occupation?/** *Usebenza phi?/* wat is jou beroep? |  | | | | | | | | | | | | | | | | |  |
| **Do you receive a grant?/** *Yayifumana imali yeGrant/* ontvang u ‘n toelae? | - Yes */ ewe/* ja | | | | | | | | | | - No / hayi/ nee | | | | | | | |
| If yes, specify/ *Ubauyaifumanacacisauthiyeyiphi/* indien ja, spesifiseer | - Child grant - *Eyo/Abantwana* - Kinder Toelaag | | | | - Pension - *Ipenshoni* - pensioën | | | | | | | | - Disability - *Eyokukhubezeka* - gestremdheid | | | | - Other - Nezinye - ander | |
| **Household Structure** | | | | | | | | | | | | | | | | | | |
| **Number of people in the household?/** *bangaphi abantu abahlala endlini/* Getalmense in U huishouding | | Children/*abantwana/ kinders*: ___________ | | | | | | | | Adults / *Bantu abadala/* Volwassenes: _______________ | | | | | | | | |
| **Family structure/** *ubume bosapho/* gesinsstruktuur | | Married/ *Nditshatile/* getroud | | Single and not living together/ *Anditshatanga kwase asihlali ndaweni nye/ enkel-lopend en woon nie saam nie* | | | | | Widowed/ *wasweleka umntu ebenditshate naye/ weduwee* | | | | | Divorced/ *Sehlukene/*geskei | | Living with extended family/ *ndihlala namanye amalungu osapho/woon met uitgebreidefamilie* | | |
| **Head of the house/**  *Intloko yekhaya/* hoof van die huis | | - Myself / *ndim/ ek* - My spouse or partner/ *Ngumntu enditshate naye/endincumisana naye/ my eggenoot* - My mother / *Ngumama/ my ma* | | | | | | - My father/ *Ngutata /* my pa - My Grandmother/Grandfather/ *NguMakhulu/Tamkhulu/ my ouma/oupa* - Member of extended family / *Lilungu losapho/* lid van uitgebreide familie | | | | | | | | | | |

1. **Responsivity and Involvement**

**Using the scale below, tick which option you think is relevant.**

*Ngokusebenzisa esiskali, tikisha apho kusondele kuwe.*

*Begruik die onderstaande skaal en merk die mees relevanste opsie*

1. **= Does not apply /** *ayikho njalo/* nie van toepassing nie
2. **= applies to me to some degree /** *injalo elohlobana / is tot n sekere mate van toepassing*
3. **= applies to me good part of the time (little more than half) /** *injalo ngamanye amaxesha (ngamaxesha angaphezu kwehafu) / is meerderheid van toepassing (net meer as die helfte)*
4. **= applies to me very much, or most of the time /** *injalo kakhulu okanye ngamaxesha onke /* is baie van toepassing, of meestal

|  | **Responsivity** | **0** | **1** | **2** | **3** |
| --- | --- | --- | --- | --- | --- |
| **1** | Do you speak to your child during the day?  *Uyathetha nomntwana ekuhambeni kwemini? (emini)*  Praat u bedags met u kind |  |  |  |  |
| **2** | Do you make eye contact when you speak to your child?  *Uyamjonga umntwana emehlweni xa uthetha naye?*  Maak u oogkontak as u met u kind praat? |  |  |  |  |
| **3** | Do you encourage other family members to speak to your child?  *Uyawakhuthaza amalungu osapho ukuba athethe nomntwana?*  Moedig u ander familielede aan om met u kind te praat? |  |  |  |  |
| **4** | Do you respond to your child when your child 'speaks' to you? (Eg. Coo's, babbles or any form of communication, or moves in the womb)  *Uye umphendule umntwana wakho xa ethetha nawe? (umzekelo xa etheketha, ethetha ulwimi lwabantwana okanye xa eshukuma ngaphakathi esiswini)*  Reageer u op u kind as u kind met u praat '? (Bv. Coo's, babbels of enige vorm van kommunikasie, of beweeg in die baarmoeder) |  |  |  |  |
| **5** | Do you encourage other family members to acknowledge and praise your child when s/he does something new?  *Uyawakhuthaza amalungu osapho ukuba mawadumise okanye avume xa umntwana enze into entsha (nivuyisane naye, nimqhwabele izandla)*  Moedig u ander gesinslede aan om u kind te erken en te prys wanneer hy / sy iets nuuts doen? |  |  |  |  |
| **6** | Do you acknowledge and praise your child when s/he does something new?  Uyavuma kwaye udumise umntwana wakho xa enze into entsha (uyamqhwabele okanye ubonise ukuba uyavuya)  Erken u en erken u u kind as hy / sy iets nuuts doen? |  |  |  |  |
| **7** | When you child wants to be held, do you respond to the child by holding him/her?  *Xa umntwana wakho efuna ubanjwa, uye uphendule ngokuthi umbambe?*  As u kind vasgehou wil word, reageer u dan op die kind deur hom / haar vas te hou? |  |  |  |  |
| **8** | When your child is naughty, do you explain to them what they did is wrong?  *Xa umntwana wakho esenza into engeyiyo, uyamchazela ukuba lento ayenzayo ayenziwa?*  As u kind stout is, verduidelik u dan vir hom/haar wat hul verkeerd gedoen het? |  |  |  |  |
| **9** | When your child does something good, do you reward their behaviour? (eg. When your child picks up their toy and packs it away, do you reward them with sweets?)  *Xa umntwana wakho enze into elungileyi, uyamnika imbuyisela kuba enze lonto? (umzekelo xa umntwana wakho echola itoyi aziqhoqhoshe, uyamnika iswiti ukubonga)*  As u kind iets goed doen, beloon u dan hul gedrag? (Bv. as u kind hul speelding optel en wegpak, beloon u hulle dan met lekkers? |  |  |  |  |
| **10** | When your child learns something new, do you encourage them (eg. Do you clap your hands to show they did a good job?)?  *Xa umntwana wakho efunda into entsha, uyamkhuthaza (umzekelo Uyamqhwabela izandla ukumbonisa ukuba wenze kakuhle)?*  As u kind iets nuuts leer, moedig u hulle aan (bv. Klap u hande om te wys dat hulle 'n goeie werk gedoen het?) |  |  |  |  |
| **11** | Do you allow your child to solve their own problems when it is safe and developmentally appropriate?  *Uhlala umvumela umntwana wakho ukuba asombulule ingxaki xa kuphephile kwaye akhule ngendlela eyiyo?*  Laat u u kind toe om hul eie probleme op te los wanneer dit veilig en ontwikkelend is? |  |  |  |  |
| **12** | Do you encourage your child’s curiosity?  *Uyamkhuthaza umntwana wakho ukuba ahlale efuna ukwazi?*  Moedig u die nuuskierigheid van u kind aan? |  |  |  |  |
|  | **Parental Involvement** |  |  |  |  |
| **13** | Do you encourage your child to play?  *Uyamkhuthaza umntana wakho ukuba adlale?*  Moedig u u kind aan om te speel? |  |  |  |  |
| **14** | Do you create playing time with your child?  *Uyalenza ixeaha lokudlala nomntwana wakho?*  Skep u tyd om saam met u kind te speel? |  |  |  |  |
| **15** | Do you make eye contact when playing with your child?  *Uyamjonga umntwana wakho emehlweni xa nidlala?*  Maak u oogkontak wanneer u met u kind speel? |  |  |  |  |
| **16** | Do you talk to your child while you are doing household work?  *Uyathetha nomntwana wakho xa usenza umsebenzi wasendlini?*  Praat u met u kind terwyl u huishoudelike werk doen? |  |  |  |  |
| **17** | Do you try to encourage your child to reach the next developmental milestone? (eg. Sitting, standing, walking, holding toys/objects with both hands)  *Uyazama ukukhuthaza umntwana ukuba makaye kwelinye inqanaba lokukhula? (umzekelo, ukuma, ukuhamba, ukubamba itoyi ngezandla zombini)*  Probeer u u kind aanmoedig om die volgende mylpaal te ontwikkel? |  |  |  |  |
| **18** | Do you encourage your partner to play with the child?  *Uyamkhuthaza umntu oncumisana naye ukuba adlale nomntwana?*  Moedig u u eggenoot aan om met die kind te speel? |  |  |  |  |
| **19** | Do you think it important for the father/mother to play with the child?  *Ucinga ukuba kubalulekile ukuba utata/umama adlale nomntwana?*  Dink u dit is belangrik dat die vader / moeder met die kind moet speel? |  |  |  |  |

*(adapted from the Child HOME inventory by Caldwell & Bradley, 1984)*

**Parental well-being**

Please read each statement and mark the number 0, 1, 2 or 3, which indicates how much the statement applies. There are no right or wrong answers.

*Ndeda ufunde ingxelo nganye wandule ukubhala isangqa kwinombola nganye 0, 1, 2, 3, echaza ukuba uvumelanga kangakanani nengxelo. Ayikho imphendulo elungileyo nengalunganga*

Lees elke stelling en kies die nommer 0, 1, 2, 3, wat aandui hoeveel die stelling van toepassing is. Daar is geen regte of verkeerde antwoorde nie.

1. **= Never /** *zange / nooit*
2. **= Sometimes /** *ngamanye amaxesha/ soms*
3. **= Often /** *oko / dikwels*
4. **= Almost always /** *ngamaxesha onke / amper altyd*

|  |  | **0** | **1** | **2** | **3** |
| --- | --- | --- | --- | --- | --- |
| **1** | I find it hard to wind down and relax  *Ndifumanisa kunzima ukwehla ndiphole.*  Ek vind dit moeilik om te ontspan |  |  |  |  |
| **2** | I was aware of dryness of my mouth  *Bendinolwazi lokoma komlomo wam*  *Ek was bewus van die droogheid in my mond.* |  |  |  |  |
| **3** | I can’t seem to experience any positive feeling at all  *Akhange ndikwazi ukuziva kamnandi.*  Dit lyk nie asof ek enigsins positiewe gevoelens ervaar nie |  |  |  |  |
| **4** | I experience breathing difficult (eg. Excessively rapid breathing, breathlessness in the absence of physical exertion).  *Ndiye ndafumanisa ubunzima ekuphefumleni (umzekelo, ukuphefumla ngokukhawuleza, ukuphelelwa ngumphefumlo ngaphandle kokusebenzisa amandle omzimba)*  Ek ervaar asemhaling moeilik (bv. Oormatige vinnige asemhaling, asemhaling in die afwesigheid van fisieke inspanning). |  |  |  |  |
| **5** | I find it difficult to work up the initiative to do things  *Ndifumana ubunzima ekwenzeni izinto ekufuneka ndizenze*  Ek vind dit moeilik om inisiatief te neem om dinge te doen |  |  |  |  |
| **6** | I over-react to situations  *Ndinokwenza izinto ngokugqithisileyo*  Ek oorreageer op situasies |  |  |  |  |
| **7** | I experienced trembling (eg. In the hands)  *Bendinokungcangcazela (umzekelo ezandleni)*  Ek het gebewe (bv. In die hande) |  |  |  |  |
| **8** | I feel that I am using a lot of nervous energy  *Ndiziva ngathi ndisebenzisa amandla amaninzi okothuka*  Ek voel dat ek baie senuweeagtige energie gebruik. |  |  |  |  |
| **9** | I am worried about situations in which I might panic and make a fool of myself  *Ndinoloyiko lwemeko ezinokuthi zindibangele uxinzelelo ndizenze isibhanxa.*  Ek is bekommerd oor situasies waarin ek paniekbevange kan raak en ‘n gek van myself maak. |  |  |  |  |
| **10** | I feel that I have nothing to look forward to  *Ndiziva ndingenanto endinothembela kuyo ekubhekeni phambili.*  Ek voel dat ek niks het om na uit te sien nie |  |  |  |  |
| **11** | I find myself getting agitated  *Ndizibhaqa ndimane ndibanomsindo ndidikiwe.*  Ek voel hoe ek geroer word |  |  |  |  |
| **12** | I feel down-hearted and blue  *Ndiye ndaziva ndinomoya ophantsi nentliziyo edandathekileyo.*  Ek voel hartseer en ongelukkig |  |  |  |  |
| **13** | I am intolerant of anything that keeps me from getting on with what I was doing  *Andinasineke yento nayiphi ethi indivimbe ekwenzeni into ebendiyenza*  Ek is onverdraagsaam teenoor enigiets wat my verhinder om aan te gaan met warmee ek besig is |  |  |  |  |
| **14** | I feel close to panic  *Ndisondele ekucaphukeni*  Ek voel naby aan paniek |  |  |  |  |
| **15** | I am unable to become enthusiastic about anything  *Bendingakwazi ukubanovuyo nokwabelana nantoni na*  Ek kan nie entoesiasties raak oor iets nie |  |  |  |  |
| **16** | I feel like I am not worth much as a person  *Bendiziva ndingexabisa njengomntu osaphefumlayo.*  Ek voel asof ek nie veel werd is as persoon nie |  |  |  |  |
| **17** | I feel that I am rather touchy  *Bendiziva ingathi ndingumntu onochuku*  Ek voel dat ek taamlik aanraak/ Ek voel taamlik gevoelig |  |  |  |  |
| **18** | I am aware of the action of my heart in the absence of physical exertion (eg. Sense of heart rate increase, heart skipping a beat)  *Ndinolwazi lwentliziyo yam nendlela ibibetha ngayo ngaphandle kosebenzisa amandla omzimba (umzekelo, ubetha kwentliziyo ngokungxamisekileyo, ukuma kwentliziyo)*  Ek is bewus van die aksie in my hart in die afwesigheid van fisieke inspanning (bv. 'n gevoel van 'n toename in hartklop, n vinniger hartklop) |  |  |  |  |
| **19** | I feel scared without any good reason  *Ndiva ndisoyika kungekho sizathu sibonakalayo*  Ek voel bang sonder enige goeie rede |  |  |  |  |
| **20** | I feel that life was meaningless  *Ndiva ngathi ubomi bam abunantsingiselo*  Ek voel dat die lewe betekenisloos was |  |  |  |  |

*(Adapted from DASS21)*

**Perceived Social Support**

We are interested in how you feel about the following statements. Read each statement carefully and indicate how you feel about each statement. There are no right or wrong answers.

*Siyarhalela ukwazi ukuba uziva kanjani ngezingxelo silandelayo. Funda ingxelo ngaye wandule ukubonisa ukuba wena uziva njani ngengxelo nganye. Akho mphedulo ilungileyo nengalulanganga.*

*Ons stel belang in hoe u oor die volgende stellings voel. Lees elke stelling versigtig en merk hoe u voel oor die stelling. Daar is geen reg of verkeerde antwoorde nie.*

1 = Very strongly disagree/ *andivumi kakhulu kakhulu / stem glad nie saam nie*

2 = Strongly disagree/ *andivumi kakhulu /* verskil sterk

3 = Mildly disagree/ *andivumi nje /* stem nie saam nie

4 = Neutral/*ndiphakathi /* neutraal

5 = Mildly agree/ *ndivuma nje /* stem bietjie saam

6 = Strongly agree/ *ndivuma kakhulu /* stem baie saam

7 = Very strongly agree/ *Ndiyavuma kakhulu kakhulu /* stem baie sterk saam

|  |  | **1** | **2** | **3** | **4** | **5** | **6** | **7** |
| --- | --- | --- | --- | --- | --- | --- | --- | --- |
|  | There is a special person who is around when I need them.  *Ukhona umntu osenyongweni okhoyo xa ndimdinga.*  Daar is ‘n spesiale persoon wat daar is wanneer ek hom/haar nodig het. |  |  |  |  |  |  |  |
|  | There is a special person with whom I can share my joys and sorrows.  *Ukhona umntu osenyongweni endingathi ndithethe naye malunga nezinto ezindiphatha kakuhle nezingandiphathi kakuhle*  Daar is’n spesiale persoon met wie ek my vreugdes en hartseer kan deel. |  |  |  |  |  |  |  |
|  | My family tries to help me.  *Usapho lwam luyazama ukundinceda.*  My familie probeer my help*. .* |  |  |  |  |  |  |  |
|  | I get the emotional help and support I need from my family  *Ndiyalufumana uncedo ngemvakakelo zam kusapho lwam*  Ek kry die emosionele hulp en ondersteuning wat ek van my gesin benodig. |  |  |  |  |  |  |  |
|  | I have a special person who is a source of comfort to me.  *Ukhona umntu osenyongweni uyindawo yam kubalekela*  Ek het ‘n spesiale persoon wat vir my ‘n troosbon is of wat vir my omgee |  |  |  |  |  |  |  |
|  | My friends try to help me.  *Abahlobo bam bayazama ukundinceda.*  My vriende probeer my help. |  |  |  |  |  |  |  |
|  | I can count on my friends when things go wrong.  *Ndingazithemba itshomi xa izinto zingahambi kakuhle*  Ek kan op my vriende staatmaak as dinge verkeerd loop. |  |  |  |  |  |  |  |
|  | I can talk about my personal problems with my family.  *Ndingathetha ngengxaki zam kusapho lwam*  Ek kan oor my persoonlike probleme praat met my vriende |  |  |  |  |  |  |  |
|  | I have friends with whom I can share my joys and sorrows.  *Ndinabo abahlobo endingathi ndithethe nabao izinto ezindonwabisayo nezingandonwabisiyo*  Ek het vriende met wie ek my vreugde en hartseer kan deel. |  |  |  |  |  |  |  |
|  | There is a special person in my life that cares about my feelings.  *Ukhona umntu osenyongweni ebomini bam ozikhathaleleyo imvakalelo zam*  Daar is ‘n spesiale persoon in my lewe wat omgee vir my gevoelens |  |  |  |  |  |  |  |
|  | My family is willing to help me make decisions.  *Usapho lwam luyafuna ukundinceda xa ndisenza izigqibo*  My familie is bereid om my te help om besluite te neem |  |  |  |  |  |  |  |
|  | I can talk about my personal problems with my friends.  *Ndingathetha malunga nengxaki zam kunye nabahlobo bam*  *Ek kan met my vriende oor my persoonlike probleme praat* |  |  |  |  |  |  |  |

*(The Multidimensional Scale of Perceived Social Support, Zimet, Dahlem, Zimet & Farley, 19*
